# Supplementary material for: Assessment of false discovery rate control in tandem mass spectrometry analysis using entrapment
Source: Nat Methods. 2025 Jun 16;22(7):1454–63. doi: 10.1038/s41592-025-02719-x (PMC12240826; doi:10.1038/s41592-025-02719-x)
Supplement: Supplementary file 2 — Reporting Summary [file 41592_2025_2719_MOESM2_ESM.pdf]

nature portfolio

Corresponding author(s): Uri Keich

Last updated by author(s): 3/17/2025

Reporting Summary

Nature Portfolio wishes to improve the reproducibility of the work that we publish. This form provides structure for consistency and transparency in reporting. For further information on Nature Portfolio policies, see our Editorial Policies and the Editorial Policy Checklist.

Statistics

For all statistical analyses, confirm that the following items are present in the figure legend, table legend, main text, or Methods section.

n/a	Confirmed	
☒	☐	The exact sample size (*n*) for each experimental group/condition, given as a discrete number and unit of measurement
☒	☐	A statement on whether measurements were taken from distinct samples or whether the same sample was measured repeatedly
☒	☐	The statistical test(s) used AND whether they are one- or two-sided<br>*Only common tests should be described solely by name; describe more complex techniques in the Methods section.*
☒	☐	A description of all covariates tested
☐	☒	A description of any assumptions or corrections, such as tests of normality and adjustment for multiple comparisons
☒	☐	A full description of the statistical parameters including central tendency (e.g. means) or other basic estimates (e.g. regression coefficient) AND variation (e.g. standard deviation) or associated estimates of uncertainty (e.g. confidence intervals)
☒	☐	For null hypothesis testing, the test statistic (e.g. *F*, *t*, *r*) with confidence intervals, effect sizes, degrees of freedom and *P* value noted<br>*Give P values as exact values whenever suitable.*
☒	☐	For Bayesian analysis, information on the choice of priors and Markov chain Monte Carlo settings
☒	☐	For hierarchical and complex designs, identification of the appropriate level for tests and full reporting of outcomes
☒	☐	Estimates of effect sizes (e.g. Cohen's *d*, Pearson's *r*), indicating how they were calculated

Our web collection on statistics for biologists contains articles on many of the points above.

Software and code

Policy information about availability of computer code

Data collection	No custom software was used to collect the data in this study.
Data analysis	Data in this study was processed and analyzed using the following software tools:<br>- Tide (within Crux v4.1.6809338)<br>- Sage (version 0.14.6)<br>- MS-GF+ (version 2023.01.12)<br>- FragPipe (version 21.1)<br>- MaxQuant (version 2.6.5.0)<br>- DIA-NN (version 1.8.1)<br>- EncyclopeDIA (version 2.12.30)<br>- Spectronaut (version 18.7.240325.55695)<br>- Oktoberfest (version 0.6.2)<br>- R (version 4.3.1)<br>Additionally, FDRBench's source code is available under the Apache 2.0 license at https://github.com/Noble-Lab/FDRBench.

For manuscripts utilizing custom algorithms or software that are central to the research but not yet described in published literature, software must be made available to editors and reviewers. We strongly encourage code deposition in a community repository (e.g. GitHub). See the Nature Portfolio guidelines for submitting code & software for further information.

Data

Policy information about availability of data

All manuscripts must include a data availability statement. This statement should provide the following information, where applicable:

- Accession codes, unique identifiers, or web links for publicly available datasets
- A description of any restrictions on data availability
- For clinical datasets or third party data, please ensure that the statement adheres to our policy

The MS/MS datasets used in this study were all downloaded from public databases. The ISB18 DDA data were download from https://regis-web.systemsbiology.net/PublicDatasets/18_Mix/Mix_7/ORBITRAP/RAW_Data/. The HEK293 DDA data were download from PRIDE with the accession number PXD001468. The DIA datasets were downloaded from Panorama, PRIDE, jPOST or MassIVE through the following accession numbers: PXD042704, PXD034525, PXD028735, PXD041421, PXD017703,PXD023325, PXD012988 and MSV000084000. The source data for reproducing the figures is available at https://doi.org/10.5281/zenodo.15073580.

Human research participants

Policy information about studies involving human research participants and Sex and Gender in Research.

Reporting on sex and gender	Not applicable
Population characteristics	Not applicable
Recruitment	Not applicable
Ethics oversight	Not applicable

Note that full information on the approval of the study protocol must also be provided in the manuscript.

Field-specific reporting

Please select the one below that is the best fit for your research. If you are not sure, read the appropriate sections before making your selection.

☒ Life sciences ☐ Behavioural & social sciences ☐ Ecological, evolutionary & environmental sciences

For a reference copy of the document with all sections, see nature.com/documents/nr-reporting-summary-flat.pdf

Life sciences study design

All studies must disclose on these points even when the disclosure is negative.

Sample size	No new data was generated for this study. All the datasets used in the study were downloaded from public databases, so the sample sizes were determined by the authors of the original studies. In this study, we selected MS runs for use to cover a single MS run scenario to multiple MS runs scenario.
Data exclusions	For the ISB18 DDA dataset, we used 9 of the 10 raw files in the present study. We excluded one raw file due to file size issue.
Replication	All the datasets used in the study were downloaded from public databases. Some of the datasets used in the study include replicates.
Randomization	All the datasets used in the study were downloaded from public databases. We selected datasets to cover different MS instruments and data types.
Blinding	Blinding is not relevant because the study focuses on method development. The study doesn't involve any new data generation.

Reporting for specific materials, systems and methods

We require information from authors about some types of materials, experimental systems and methods used in many studies. Here, indicate whether each material, system or method listed is relevant to your study. If you are not sure if a list item applies to your research, read the appropriate section before selecting a response.

Materials & experimental systems

n/a	Involved in the study	
☒	☐	Antibodies
☒	☐	Eukaryotic cell lines
☒	☐	Palaeontology and archaeology
☒	☐	Animals and other organisms
☒	☐	Clinical data
☒	☐	Dual use research of concern

Methods

n/a	Involved in the study	
☒	☐	ChIP-seq
☒	☐	Flow cytometry
☒	☐	MRI-based neuroimaging
